# Strategies for optimizing clinical trial recruitment: perspectives among patients with breast cancer

**DOI:** 10.1007/s10552-026-02140-5

**Published:** 2026-02-19

**Authors:** Mandy Chen, Nicole Reh, Sindhu R. Dwarampudi, Courtney P. Williams, Nicole Henderson, Gabrielle B. Rocque, Lily Gutnik

**Affiliations:** 1 Heersink School of Medicine, University of Alabama at Birmingham, 1670 University Blvd, Birmingham, AL 35233 USA; 2 Medical College of Georgia, Augusta, USA; 3 Division of General Internal Medicine & Population Science, Birmingham, USA; 4 O’Neal Comprehensive Cancer Center, Birmingham, USA; 5 Division of Hematology & Oncology, University of Alabama at Birmingham, Birmingham, AL USA; 6 Department of Surgery, University of Alabama at Birmingham, Birmingham, AL USA

**Keywords:** Breast cancer, Clinical trial recruitment, Clinical trial enrollment, Patient perspective

## Abstract

**Purpose:**

Despite the critical role of clinical trials in advancing cancer treatment, patient enrollment remains low at less than 10% with various health system-, provider-, and patient-level barriers [1]. This study explored perspectives of patients with breast cancer receiving treatment who declined clinical trial participation to work towards developing more effective, patient-centered recruitment strategies.

**Methods:**

This qualitative study enrolled patients who were offered participation in a breast cancer clinical trial yet declined to participate from August 2023 to March 2024. Semi-structured interviews were used to elucidate patient perspectives on the clinical trial recruitment process. An open coding scheme was used to identify major themes present throughout the transcribed interviewers. Two independent reviewers then coded using NVivo 14 software with a third reviewer to settle any discrepancies.

**Results:**

Of the 21 female patients interviewed, 43% had previously declined enrollment onto a therapeutic clinical trial and 57% a non-therapeutic clinical trial. Three core themes regarding patient-suggested strategies to optimize clinical trial recruitment emerged: (1) improving communication with patients (e.g., ensuring presentation of key information, timing of recruitment, manner of information delivery, provider involvement), (2) increasing accessibility/availability of resources to address logistical burdens (e.g., ride-share, telehealth, compensation), and (3) leveraging media in recruitment platforms.

**Conclusions:**

This study highlights critical areas for optimization of clinical trial recruitment offering strategies such as the use of lay patient navigators, physician/researcher recruitment training in communication and implicit biases, and mixed/online recruitment. This feedback can better inform more patient centric recruitment strategies.

## Introduction

Despite the importance of clinical trials in advancing cancer care, less than one in ten patients with cancer participate in clinical trials due to health system-, trial-, provider-, and patient-level barriers [1]. Recruitment and enrollment of participants to clinical trials is a multi-step process which involves identifying patients that meet eligibility criteria, presenting the study to potential participants, allowing them to review the information to make a decision about participation, and subsequently obtaining informed consent if they agree to enroll in the study. Multiple patient-level barriers contribute to low rates of enrollment including mistrust of research and the medical system, perceived harms, costs of participating, availability of transportation, lack of knowledge on clinical trials, time commitment, and fear [2]. Socioeconomic disadvantage indicated by high ADI, low education, low income, lack of insurance coverage, and poor social support is consistently associated with lower clinical trial access and participation [3–6]. Rurality, race, and ethnicity are amongst other additional factors that effect trial invitation, availability, and willingness to participate [4–7]. These barriers are inequitably high, especially for patients in the Deep South (LA, MS, AL, GA, SC), where socioeconomic, racial, and geographic factors predispose to higher prevalence of medical mistrust, lower digital health literacy, deficient access to internet coverage, and lack of adequate transportation [8–16]. The primary reason clinical trials fail to complete is attributed to poor accrual [17]. This lower rate of recruitment is unfavorable as clinical trials that have higher enrollment rates report prolonged survival, mortality reductions, and produce treatment advances at a faster rate [18]. Additionally, failure to recruit an adequate and diverse amount of participants reduces the statistical power of the study and, thus, reduces the inferential ability of the study data.

Various strategies can be implemented to optimize the recruitment process and mitigate these barriers to enrollment. Existing patient-level interventions targeting patient-level barriers to increase clinical trial enrollment and optimize recruitment range from addressing socioeconomic barriers such as payments distributed to participants, coverage of travel expenses and meals, recruitment campaigns, and patient advocates [18, 19]. However, little is known about the perspectives of patients who reside in the Deep South who have been offered, yet, declined clinical trials and their thoughts on how to optimize the recruitment process [20, 21]. Here we present findings from semi-structured interviews with patients with breast cancer who were eligible yet declined a clinical trial, assessing their perspectives on the clinical trial recruitment process and their suggestions on increasing patient enrollment.

## Methods

### Study design and sample

This cross-sectional qualitative study was conducted from August 2023 to March 2024, exploring patient perspectives on clinical trial participation through semi-structured interviews at University of Alabama at Birmingham Hospital, a National Cancer Institute-designated Comprehensive Cancer Center in the Deep South. Eligible participants were women with breast cancer that were eligible for and offered any type of breast cancer clinical trial, therapeutic or non-therapeutic, between July 2020 and January 2023 and declined to participate. These patients were identified through an internal database that tracks patients with breast cancer who were referred to a clinical trial. Eligibility, initial screening, and recruiting for clinical trials was determined by patients’ medical, surgical, or radiation oncologist or through discussion at a multidisciplinary treatment planning meeting with consideration of various criteria such as cancer subtype and stage [22]. Each patient was then screened by study coordinators per each study’s specific criteria [22]. Recruitment of eligible participants for this study was done both in-person at patient clinic visits and through phone calls, with participants receiving $25 compensation for participation in the study—the amount of compensation determined to be fair, reasonable, and proportional to time and effort given to participate in this study. Compensation was provided regardless of participant interview questions responses, and patients were informed they could stop the interview at any time. To ensure adequate sample size, thematic saturation, specified as no new emergent themes, was assessed throughout data collection [23]. This study was approved by the UAB Institutional Review Board (IRB-300001910). Methods and findings were reported following the Consolidated criteria for reporting qualitative studies (COREQ) 32-item checklist [24].

### Setting and data collection

Interviews that were approximately an hour long were conducted by a research assistant (NR; female) who had no prior relationship with the patients via Zoom using a semi-structured interview guide developed by the research team. Patients were informed of the general study goals to understand patients’ thoughts and perspectives on clinical trials and their decision to participate or decline. The interview guide was developed by members of this research team with clinical context and methodological expertise (LG, GR, NH, CW, all female) focused on barriers and facilitators to clinical trial participation with questions accessing patients’ thoughts on clinical trials, personal experiences with clinical trials participation, if any, and the contribution of commonly cited factors including finances, fears, side effects, transportation on patients’ decision to enroll in clinical trials. Prior to beginning the interview, patients were verbally consented. To mitigate power imbalances, participants were informed that there were no right or wrong answers, participation was voluntary, and confidentiality/deidentification was emphasized. The interviews were then audio-recorded and transcribed verbatim by an independent transcription service. Patient demographic information, including age, race, ethnicity, home address, marital status, religion, and insurance status were extracted from the electronic medical record. Patient home address was used to calculate Area Deprivation Index, a geographic area-level measure of neighborhood disadvantage used as a proxy measure for socioeconomic status [25]. Previous trial participation was self-reported and abstracted from the internal clinical trial referral database. No additional field notes were taken during or after interviews nor were any repeat interviews held. Transcripts and findings were not returned to participants for feedback.

### Data analysis

An open coding scheme was first developed by the breast surgical oncologist (LG; female) who identified major themes present throughout the transcribed interviews using content analysis approach. Transcribed interviews were then coded inductively by two independent reviewers (NR, MC; female), using the original open code. The independent reviewers (NR, MC; female) met consistently after about every three to four coded transcripts to maintain continuity and consensus, using a third reviewer (LG) to settle any discrepancies. Intercoder reliability was analyzed using the kappa statistic and determined to be acceptable (k = 0.73). Emergent themes, patterns, and quotes were identified using a content analysis approach. All data were analyzed using NVivo V14 software.

## Results

Of 120 individuals with whom contact was attempted, 36 initially agreed to participate in our study with 21 ultimately completing interviews, 6 were deceased, and 78 either did not answer or declined due to reasons such as having just finished another clinical trial, having to take care of sick family members, being busy, feeling sick, not interested in the study, wanting to focus on family, not wanting to think about clinical trials, or difficulty speaking.. Of the 15 participants who initially agreed to participate, 7 did not attend their scheduled interviews and did not respond to follow-up phone calls; 5 requested that interviews be rescheduled but did not respond to subsequent outreach (two follow-up calls with voicemails left for each); 1 missed an initial interview, rescheduled, and then did not respond to further contact; 1 agreed initially but later declined participation after consulting with family members; and 1 missed the scheduled interview and declined participation when contacted to reschedule either due to being lost to follow-up, scheduling conflicts, or other unspecified reasons. Twenty-one female patients with breast cancer were included in the study, 43% (*n* = 9) of which previously refused a therapeutic clinical trial and 57% (*n* = 12) of which previously refused a non-therapeutic clinical trial. The mean age was 57 (SD 12), 48% were White, 52% were married, 33% lived in a highly deprived neighborhood, 5% lived in rural areas,76% reported a religious affiliation, and 52% had commercial insurance. Over half (52%, *n* = 11) of participants had previously participated in a clinical trial, while 43% (*n* = 9) had never participated in a trial and 8.33% (*n* = 1) did not disclose prior clinical trial participation (Table 1).

**Table 1:** Sociodemographic characteristics of interviewed patients

	Therapeutic (*n* = 9)		Non-Therapeutic (*n* = 12)		Total (*n* = 21)	
	*n*	%	*n*	%	*n*	%
*Age*	54	SD = 12.42	60	SD = 11.79	57	SD = 12.10
*Race*						
White	6	66.67	4	33.33	10	47.62
Black	2	22.22	7	58.33	9	42.86
Asian	0	0	1	8.33	1	4.76
Decline to answer	1	11.11	0	0	1	4.76
*Ethnicity*						
Non-Hispanic/Latino	8	88.89	11	91.67	20	90.9
Unknown	1	11.11	1	8.33	2	9.1
*Area deprivation index*						
Low	7	77.78	7	58.33	14	66.67
High	2	22.22	5	41.67	7	33.33
*Residence type*						
Urban	8	88.89	12	100	20	95.24
Rural	1	11.11	0	0	1	4.76
*Marital status*						
Married	6	66.67	5	41.67	11	52.38
Single	2	22.22	7	58.33	9	42.86
Widowed	1	11.11	0	0	1	4.76
*Religion*						
Christian	4	44.44	12	100	16	76.19
None	4	44.44	0	0	4	19.05
Unknown	1	11.11	0	0	1	4.76
*Insurance*						
Commercial	7	77.78	4	33.33	11	52.38
Medicaid	1	11.11	3	25	4	19.05
Medicare	1	11.11	6	50	7	33.33
*Previous trial participation*						
Yes	5	55.56	6	50	11	52.38
No	4	44.44	5	41.67	9	42.86
Unknown	0	0	1	8.33	1	4.76

**Fig. 1:**
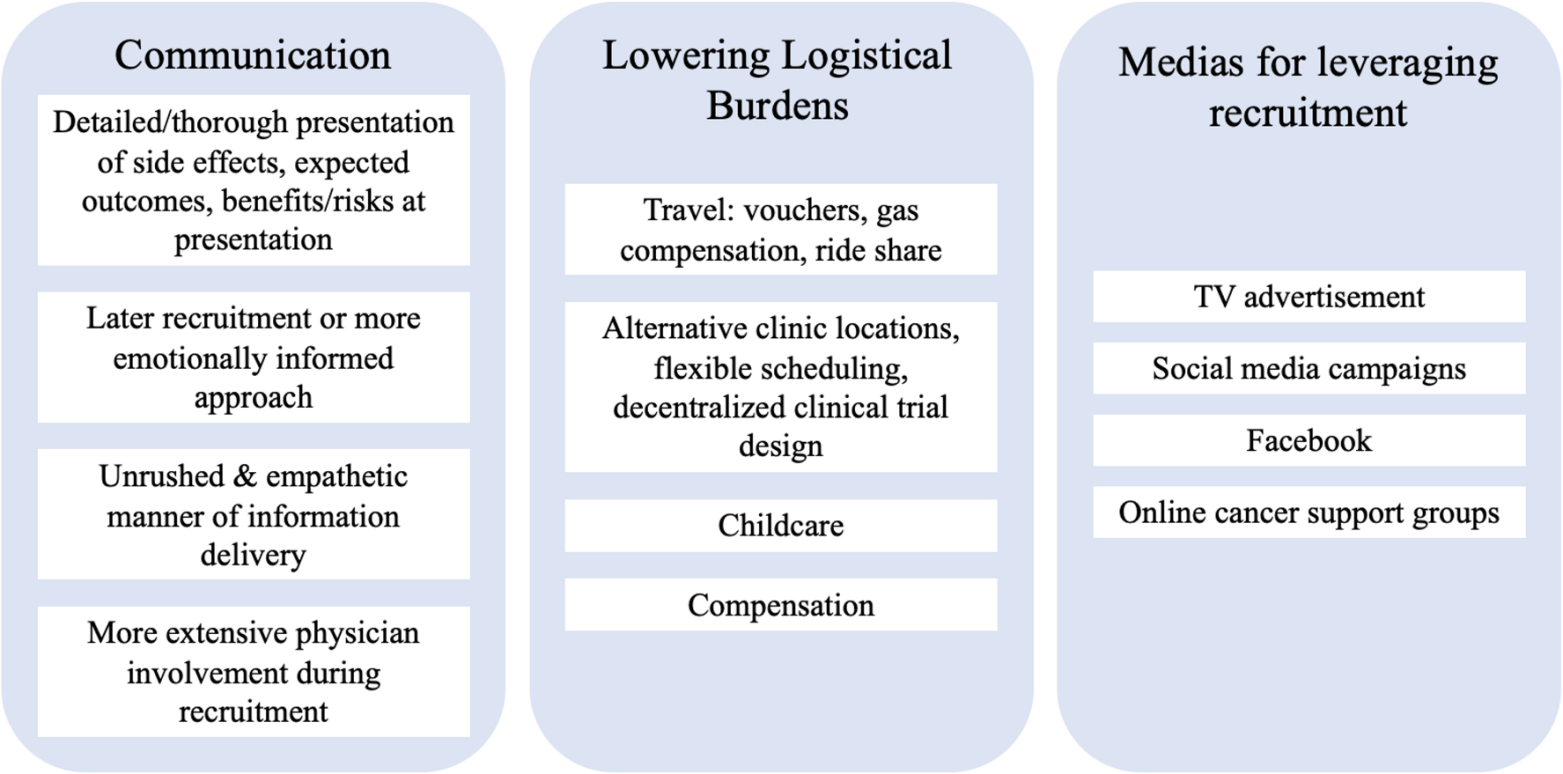
Patient suggested strategies for optimizing clinical trial recruitment

Major themes regarding increasing trial participation and optimizing trial recruitment from interview participants largely centered on (1) improving communication with patients, (2) increasing accessibility/availability of resources to address logistical burdens, and (3) leveraging media in recruitment platform (Fig. 1).

### Communication

#### Timing of recruitment

Many patients (38%) reported that they were recruited from clinical trials during their initial physician visit or chemotherapy appointments and early on in their breast cancer diagnosis which many found to be emotionally overwhelming and inappropriate, as one patient explained, “*It was during one of my chemo treatments. And like I said, at the time, I was already sick. That was my very first chemo treatment. I remember someone coming around and asking me if I wanted to participate in a clinical trial, and that was just not the right time. It was just not the right time. You are already sitting in the chair, you got fixing to get ready to get drugs pumped through you, and you don’t know what kind of side effects you’re going to have from it. And just to me, offering clinical trials at the beginning of a chemo treatment is just not the right…I just thought the timing was inappropriate*” (P14). Similarly, another patient expressed, “*I don’t even remember, but I was just totally was not interested…they caught me in a bad timing possibly because I was very emotional around that time. So it was kind of like they didn’t do it the right way”* (P17).

#### Information presented at recruitment

Almost all (90%) of patients emphasized a need for better communication during the recruitment process either having experienced good communication or finding it lacking in their previous experiences with recruitment meetings. Most patients (81%) mentioned the need for thorough, clear explanations about trial’s logistics, side effects, goals/expected outcomes, benefits, and risks, which they expressed was crucial in making informed decisions. One patient stated, *“I think communication is definitely a key factor there as far as telling people about it and giving them the information and where else they may go look for information”* (P20) and many others similarly expressed the importance of explaining *“the percentage of the good outcome versus the bad outcome”* (P1), *“what the side effects were and what the chances are of things not going well”* (P2) and *“how often you have to be here, what’s involved, or tissue, blood, just whatever. I think all that need to be exposed at that time and not surprise them later on”* (P5). Three patients discussed the importance of being given educational resources where patients could further obtain information on the trial and be more informed in making their decision.

#### Manner of information delivery

Patients emphasized the manner of information delivery by the provider as a crucial factor in their decision to participate or refuse clinical trials, with some stating, “*You can see it on their face, that they’re frightened, and you begin to talk down to them. Like…I’ve got all the answers. Well, no, you don’t. That can scare people away from a clinical trial…So I think a lot of doctors need to be aware of their presentations”* (P1). Another patient stated *“I have to feel that you care enough to sit here and talk with me about what you need me to do to help other people. And if you’re invested, you can tell the people who are invested in their jobs and who care about what they’re doing”* (P15). Many patients (43%) felt that the manner of how they were approached about clinical trials was unrushed and respectful of patient autonomy, making them feel comfortable in their decision-making process. Patients expressed, “*He gave us options. We could definitely do the mastectomy, we could do the regular chemo, we could do the clinical trial. And he didn’t say we had to make a choice right then and there. He gave me time to talk it over, think about it”* (P6).

#### Patient-provider relationship

Approximately half of the patients focused on the physician–patient interaction and its importance in recruiting patients for clinical trials. Two patients (9.5%) stated having their doctors physically present to answer questions during the recruitment process would facilitate their decision to participate in clinical trials, while others encouraged physicians to take a more personal, less disconnected role in the decision-making process. Specifically, one patient (4.7%) suggested doctors communicate with patients as though they were a friend or family member, with one stating, *“I’m sure most people want the doctor’s opinion. What would you do if this were your wife or your mother or whatever? Some doctors don’t want to do that, but that’s helpful”* (P9). Some patients (38%) stated this was due to the trust they had in their healthcare providers as they perceived them to be caring and knowledgeable. *“I think you would pay more attention to it coming from your doctor than you would from just seeing it on a news report or something like that”* (P11) Specifically, a patient noted, “*I prefer my doctor to be either present or he have a say-so…even though I make my own decision about me participating in a clinical trial, I would want him to have a say-so, or input…Because he would know”* (P17), describing how doctors could impact the decision to enroll in clinical trials in a more nuanced manner due to their knowledge on how trial treatment may specifically affect them or how to mitigate some minor side effects that could otherwise deter patients from participating.

The type of provider present during the clinical trials was mentioned as having an impact on decision whether to participate in clinical trials. One patient expressed that they felt physicians should be more readily available during a clinical trial to provide reassurance or answer questions, stating, *“I think for a clinical trial, I think the physician should be on hand to give that patient some immediate answer…I think physicians should be more accessible during a clinical trial”* (P1). Other patients stated, *“I would think they would talk to their doctor…When he told me about it, I didn’t hesitate one bit because he’s the one giving it. I just didn’t feel like my doctor would ever give me anything that would hurt me because he was my doctor.”* (P16), expressing the value they place in the medical opinion of physicians, and therefore, the physician endorsement of clinical trials acted as a facilitator to clinical trial participation.

### Addressing logistical burdens of trial participation

Patients suggested availing resources that could aid in access to, and convenience of the trial known during the recruitment discussion. These included a variety of suggestions to address the myriad of logistical burden on participants because *“if you live outside of a certain mile radius for your time to come here for extra gas money and stuff…I mean it’s a lot, I mean it’s a lot, a lot, a lot, a lot”* (P7). Most (52%) mentioned solutions regarding transportation such as gas vouchers or rideshare coverage to and from appointments and having “*someone pick them up*” (P9) as travel, a well-known barrier to clinical trial participation, could weigh heavily in the initial decision-making process, and therefore, should be addressed early on in the recruitment process. Moreover, scheduling details were found to be an important component within the recruitment discussion with patients proposing offering alternative clinic locations and more flexible scheduling. Patients suggested the use of branch clinics or other affiliated medical facilities closer to patients’ residence and the use of *“Zoom type things that can be utilized like telehealth”* (P18) whenever possible to reduce travel burden of getting to trial-related appointments. Another patient suggested *“start a nursery in the hospital, let them keep their kids there”* (P9) to address logistical barriers beyond patient mobility or travel as difficulty obtaining childcare services was mentioned several times as a barrier to clinical trial participation.

#### Compensation

Most patients (76%) suggested compensation as a means of increasing clinical trial participation; however, two patients believed compensation could possibly decrease participation. Most (76%) believed any sort of compensation or incentivization, financial or otherwise, would increase trial participation by offsetting *“expenses for doing this trial”* such as *“what they would lose at work that day or what it would cost them to hire a babysitter”* (P9) and *“if they’re not paid for taking off”* (P13). Many of the suggestions regarding compensation included stipends, food, gas, and parking. Two patients expressed compensation serves as a tool to demonstrate care and appreciation for patients’ time and energy invested in a clinical trial, with one patient stating, *“It’s almost like a token of appreciation for you to do this for us”* (P13) and another suggesting to *“bump in a little bit more just to show the appreciation”* (P8).

### Leveraging media in a recruitment platform

Patients mentioned the frequency of exposure to clinical trial information *“for those people who are able to see TV, especially these local channels…they have an opportunity to constantly hear about clinical trials…commercials made it comfortable because I was accustomed hearing about clinical trials”* (P5) and *“you see all these commercials for St. Jude…If I’m a parent in a situation like that, then I’m all excited to be able to go to St. Jude”* (P1). Two patients mentioned that social media outlets were a *“really great source because it’s available online”* (P18) and could present effective opportunities for physicians to communicate about clinical trials. *“I would say the social media…you’re not going to hear about it from your doctor”* (P18) they said describing how they would be more likely to learn about clinical trials through social media versus at a busy clinic visit with their physician. Facebook, in particular, was discussed as being a place where many people may discover clinical trials with a patient remarking *“you can even see some on Facebook nowadays for smoking. I see that a lot on Facebook right now”* (P6). Moreover, there exists a surplus of breast cancer online support groups for patients on that platform. *“I think that it might be a good idea to go in saying, “I don’t have cancer, but I am working in research, and we’re educating ourselves,”* (P18) one patient suggested researchers/physicians joining online groups, reasoning that physician or medical expert presence on these forums could be a valuable opportunity for them to reach patients and increase health literacy regarding clinical trials.

## Discussion

Recruitment and patient enrollment often pose a significant challenge within clinical trials especially within the Deep South due to various factors such as medical mistrust, low health literacy, or geographic barriers to trial participation leading a subset of patients underrepresented in trials and non-generalizable results. This study revealed key themes from the perspectives of patients with breast cancer who declined clinical trial participation, a unique and difficult to reach population therefore providing unique and valualbe insight. Improving communication with patients, addressing logistical burdens of trial participation, and use of social media as a recruitment platform emerged as critical areas for optimizing clinical trial recruitment strategies. Moreover, this study’s particular focus on the residents of the Deep South, an underserved population, also aligns with ASCO, NCI, and NIMHD’s priorities regarding efforts to increase equitable trial access [26].

### Patient communication about trials

Physicians play a major role in clinical trial recruitment with studies reporting that 76% of patients expected physicians to alert them to any appropriate clinical trials [27, 28]. In our study many patients reported wanting their physicians to be the ones conducting the recruitment as they valued the trust and continuity of care within the established patient-physician relationship. This aligns with other studies that find physician referral as one of the most useful recruitment strategies and that patients prefer to be recruited by their physician, specifically primary care and family medicine physicians due to trust [29–33]. One such study found women at high risk of breast cancer were 13 times more likely to participate in a chemoprevention trial when advised to by their primary physician [32]. Physician involvement is key especially in rural communities where patients often see their one provider who they have an established relationship of trust with, facilitating discussion of and dispelling skepticism towards clinical trials more seamlessly. Additionally, physicians already have extensive experience conducting difficult and sensitive conversations with patients, whereas, recruiters have less interactions and a shorter duration to spend with patients to gain their trust [34]. Furthermore, training programs such as the Just ASK initiative allow physicians to be more aware of implicit biases that affect who they do or do not choose to bring up the topic of clinical trials for with goal of having physicians comfortably bring up clinical trial participation among all patient types [35].

Goals of therapy, outcomes, and possible side effects were topics important to patients to discuss during recruitment meetings. Consistent with other studies, our patients believed unrushed and clear communication of this information in a manner respectful of patient autonomy to be crucial during the recruitment process [36]. However, this may not always be the case due to time constraints within the clinic. The use of clinical trial lay navigators could be a useful way to address patient concerns of recruitment meetings being rushed or not having enough time to review trial details. Patients enrolled in patient navigation programs have low clinical trial refusal rates of 4–6% [37]. Most patients will accept the use of lay navigators and will be satisfied with their services, though some patients that may not face specific barriers to trial participation may decline use of them [19, 37–39]. The application of navigators in community locations allowed for better reach of clinical trial recruitment in minority patient populations [38]. Video technology could be utilized in teaching for various trial-related topics while simultaneously addressing concerns of recruitment being “rushed,” giving potential participants more resources.

Communication during initial recruitment is vitally important, however, studies show that the quality of this initial discussion with patients regarding clinical trials is highly variable and many researchers lack training on how to talk to people about clinical trials [40]. Positive attitude of providers and research staff towards clinical trial was a non-verbal aspect of communication patients found to be important during the recruitment process which aligns with findings of other studies [41, 42].

Clinical trial recruitment was found to occur early on in patients’ diagnosis and often during difficult clinic or treatment (chemotherapy) appointments, similar to previous literature which was both emotionally and physically tolling. Use of online recruitment strategies especially in non-therapeutic clinical trials that may have less urgency/impact on patients’ care could also aid in decreasing the emotional and physical toll of being kept up at appointments as it could allow potential participants to consider clinical trials at a time appropriate for themselves and be a source for self-directed learning about the clinical trial. However, this strategy could be limited due to the scarcity of internet and technology access within communities such as the Deep South or the Alabama Black Belt region and by low health literacy which is especially relevant to patients served by our health system.

While discussion of clinical trial may be limited due to clinic time constraints or lack of time for physicians to learn the intricacies of various trials, physicians could still be peripherally involved in recruitment activities such as being present during recruitment or prefacing the recruitment visit of research staff to aid in establishing trust between their patient and recruiters. Studies have also found that introduction of a clinical trial and research staff by physicians helped build subsequent rapport with patients [41].

### Logistical burdens to be addressed during recruitment

Using navigators to connect patients with suitable trials that accounted for barriers to participation is an effective means to mitigate any social determinant of health factors that could present barriers to participating in clinical trials [38]. Thus, introduction of navigators early during the recruitment process could be beneficial in addressing such logistical barriers. As these barriers are considerable, efforts such as the Austrial Teletrial Program could help alleviate disproportionate underrepresentation of such patients by utilizing telecommunications technology and multiple sites in a decentralized design to improve access [43, 44].

### Media as recruitment strategy

Our study found that online social media outlets, online forums, and, specifically, Facebook could potentially be a good target for clinical trial recruitment campaigns. One study found social media recruitment to be an especially effective tool,increasing accrual by eight-fold, particularly, with the use of Facebook as our study suggests [45]. Moreover, social media use in recruitment yielded greater diversity of income, employment status, cancer type, and geography while proving to be substantially more cost effective [45]. Facebook and other social media platforms use algorithms that aim to show more content they think users will be interested in based on user engagement with said content. Therefore, online recruitment can be more efficient in that clinical trial information would get self-populated by the individuals interested in learning more about that study, eliminating barriers of recruitment such as unsolicited approaches to potential participants to gauge interest. There exists many online support groups and forums, but oftentimes, there is no healthcare professional present other than if the patients, themselves, work within the healthcare field. Therefore, use of these online forums and media, especially, Facebook could optimize trial recruitment. Moreover, studies show that Facebook is often the social media of choice for use in online recruitment strategies as 71% of US adults that use social media use Facebook [18].

In some geographic areas, there is poor cellular service, lack of internet, or devices able to connect to the internet. Specifically, within Alabama’s 24 Black Belt counties, all but 2 are below the statewide average of 86% for broadband internet coverage with less than half able to access an affordable internet plan defined as $60 or less per month and computer access at a much lower rate of 77.5% than state and national rates of 54.2 and 91.9%, respectively [12–16]. This and patients with low e-health literacy introduces the issue of the use of online recruitment strategies potentially alienating potential participants [46, 47]. However, with the rise of the internet, older participants recruited through Facebook surpassed traditional recruitment methods [48]. There is no association in recruitment rates with any one particular recruitment strategy, whether offline or mixed, but mixed methods are more efficient than just offline strategies alone [49]. Therefore, online recruitment strategies used in combination and supplementary to primary care referrals and other means of recruitment could offer more flexibility and choices to enable more diverse participation, improve inclusivity, and recruit participants that reflect real world demographics [49, 50].

### Limitations

This study was a unique opportunity to learn and develop strategies based on the input of patients who were offered and declined trial participation, however, the exploratory and hypothesis generating nature of qualitative research introduces inherent limitations. With a maximum gap of 4 years between when patients declined clinical trial (2020–2023) and when patients were interviewed (2023–2024), there could be a concern for recall bias; however, as the study was not investigating experience with that specific timepoint of clinical trial recruitment but rather any and all past experiences with clinical trial recruitment in general, any effect of time should be minimal. This was a single center study at an academic medical center in the Deep South which may limit generalizability. Patient demographics were reflective of racial and socioeconomic demographics in the Deep South which may be less reflective of other populations. Additionally, this study only examined women with breast cancer, other patients with different diagnoses, options, and backgrounds could have differing viewpoints and suggestions. While information regarding therapeutic or non-therapeutic subgroups and other demographic subgroups were gathered, subgroup analysis with the themes was not conducted given the small number of participants in each category. Further studies in other patient populations are required to assess generalizability in other clinical trial fields.

## Conclusion

This study offers valuable insights into optimizing clinical trial recruitment by incorporating feedback directly from a unique population of patients from the Deep South who were eligible to participate in and offered a trial, yet declined. We highlight critical areas for optimization of clinical trial recruitment of women with breast cancer, underscoring the delivery of relevant information, appropriate timing of recruitment, and the way information is presented. This feedback can better inform more patient centric recruitment strategies for clinical trials, reflecting the components of recruitment that underrepresented patients find important. These results mostly support existing literature regarding clinical trial recruitment strategies; however, future efforts to optimize clinical trial recruitment should focus on determining if there are any significant differences between any particular strategies and establishing guidelines for compensation.

## Data Availability

The data that support the findings of this study are available on request from the corresponding author. The data are not publicly available due to privacy or ethical restrictions.
